# Icariin, a flavonoid with anti-cancer effects, alleviated paclitaxel-induced neuropathic pain in a SIRT1-dependent manner

**DOI:** 10.1177/1744806918768970

**Published:** 2018-04-06

**Authors:** Yulong Gui, Jie Zhang, Liang Chen, Shunyuan Duan, Jing Tang, Wei Xu, Aiyuan Li

**Affiliations:** 1 Department of Anesthesiology, Hunan Provincial Maternal and Child Health Hospital, Changsha, China; 2 Department of Endocrinology, Yongzhou-Affiliated Hospital of University of South China, Yongzhou, China

**Keywords:** Icariin, paclitaxel-induced neuropathic pain, spinal neuroinflammation, SIRT1, NF-κB(p65)

## Abstract

**Background:**

One of the most common side effects of paclitaxel was dosage-dependently painful neuropathy. Various reports indicated that spinal neuroinflammation was involved in paclitaxel-induced neuropathic pain. This study investigated the effect of icariin on paclitaxel-induced neuroinflammation and peripheral neuropathy in rats.

**Methods:**

Two parts were included in this study. In part one, the effect of icariin on paclitaxel-induced neuropathic pain was investigated. Mechanical thresholds were measured as primary outcomes. Production of proinflammatory factors (tumor necrosis factor-α, interleukin-1 β, and interleukin-6), activation of nuclear factor-κB (NF-κB(p65)) signal, and activation of astrocytes were detected as secondary outcomes. Spinal Sirtuin 1 (SIRT1) expression, H4 acetylation, and NAD^+^ content were measured to investigate the effect of icariin on spinal SIRT1 signal pathway. In part two, the role of SIRT1 signal on icariin-induced effect in rats was investigated, and EX527, a SIRT1 inhibitor, was employed.

**Results:**

The results showed paclitaxel treatment induced significant decrease in mechanical thresholds. Paclitaxel treatment also induced NF-κB(p65) activation and upregulation of proinflammatory factors (TNF-α, IL-1β, and IL-6). Paclitaxel also induced astrocyte activation in the spinal cord. However, 100 mg/kg icariin treatment significantly alleviated paclitaxel-induced mechanical allodynia and spinal neuroinflammation. Furthermore, icariin treatment dosage-dependently reversed paclitaxel-induced SIRT1 downregulation and H4 acetylation. EX527, a selective SIRT1 inhibitor, completely reversed icariin-induced anti-neuroinflammation and anti-allodynia effects in paclitaxel-induced neuropathic pain rats.

**Conclusions:**

This meant that spinal SIRT1 activation was involved in icariin-induced effects in paclitaxel-induced neuropathic pain rats. Icariin could be a potential agent for the treatment of paclitaxel-induced neuropathic pain.

## Introduction

As a widely used chemotherapeutic drug for the treatment of solid tumors, one of the most common side effects of paclitaxel was dosage-dependently painful neuropathy. Paclitaxel-induced neuropathic pain (PINP) affected the quality of patients’ daily life and lacked effective treatment. Researches on potential agents were still needed.

Various studies proved that spinal neuroinflammation was involved in the formation of PINP. Li et al. reported that toll-like receptor 4/NF-κB(p65) signaling promoted development of PINP.^1,2^ Li et al.^3^ reported that NF-κB(p65)-dependent histone 4 (H4) acetylation promoted the production of pro-inflammatory factors such as CX3CL1. However, direct inhibition of NF-κB(p65) signal reduced chemotherapy-induced reactive oxygen species production and attenuated the effect of the therapy. So, H4 acetylation could be a potential target for the treatment of PINP.

SIRT1, or Sirtuin 1, is a histone deacetylase that regulates various physical activities. It is activated in NAD-dependent and NAD-independent ways.^4^ It was reported that spinal SIRT1 activation decreased H4 acetylation (especially, H4-16K point) and relieved kinds of neuropathic pain. Nicotinamide riboside, as a NAD^+^ precursor, also alleviated PINP in female rats.^5^ However, resveratrol, a SIRT1 selective agonist, showed different effects on different kinds of solid tumors. Rigolio et al.^6^ reported that resveratrol protected human neuroblastoma SH-SY5Y cell from paclitaxel-induced apoptosis.

Icariin, a flavonoid extracted from Epimedium brevicornum Maxim, possessed anti-inflammation, male enhancement, and anti-cancer effects.^7^ Recent studies showed that icariin reduced lipopolysaccharide-induced neuroinflammation by decreasing NF-κB(p65) activation.^8^ Besides, icariin was reported to protect against brain injury through SIRT1 signaling.^9,10^ This prompted us to investigate the effect of icariin on PINP and the role of SIRT1 in icariin-induced effects in PINP rats.

In this study, mechanical thresholds (MTs) were measured as a primary outcome. Western blot, enzyme-linked immunosorbent assay (ELISA), and immunofluorescence results for neuroinflammation were treated as secondary outcomes. To explore the mechanism in icariin-induced effects, EX527 (Selisistat), a selective SIRT1 antagonist, was used in this study.^11^

## Methods

### Subjects

Three- to four-month-old male Sprague Dawley rats weighing 220 to 250 g were used in this study. All the experiments and procedures were approved by the Animal Care and Use Committee of University of South China (Hunan, China). Rats were housed in a homothermal environment (22–25°C) with a 12/12-h light-dark cycle and access to water and food freely. The experiments were performed after 1 week of acclimatization.

### Groups and treatments

There were two parts in this study. In part one, rats were randomized into seven groups (*n*=12): Control, Control+icariin 100 mg/kg, PINP, PINP +vehicle, PINP+icariin 25 mg/kg, PINP + icariin 50 mg/kg, and PINP + icariin 100 mg/kg group. Half of the rats in each group were used for behavior assessment. MTs were tested on day 8, 11, 15, 19, and 22. On day 8, the MTs were measured at 1 h before and 1, 3, 5, and 7 h after icariin administration. The other half were sacrificed in deep anesthesia for NAD^+^ measurement, ELISA, immunoblot, and immunohistochemistry tests at 1 h after icariin on day 15. In part two, rats were randomized into five groups (*n*=12): PINP + icariin 100 mg/kg, PINP +icariin 100 mg/kg +vehicle group, PINP + icariin 100 mg/kg+ EX527 group, control + EX527 group, and PINP +EX527, PINP group. Behavior tests were performed in six rats in each group. The other half were killed in deep anesthesia for immunoblot results.

### Drug administration

In part one, icariin (Shanghai CIVI Chemical Technology Co., China, 98%), dissolved in ethyl acetate, was intragastric administrated from day 8 to day 15. The dosage of icariin was performed according to previous study in neuroscience^9^ and Meeh-Rubner formula. Vehicle rats were treated with ethyl acetate. In part two, EX527 was intrathecally administrated with icariin administration, simultaneously. The dosage of EX527 was according to previous study.^12^ Five microliters of 1.2 mM EX-527 in 20% dimethyl sulfoxide was administrated from day 8 to day 15. Vehicle rats were treated with 20% dimethyl sulfoxide.

### Intrathecal administration

In part two, rats were treated with intrathecal catheter placement 2 weeks before the experiments started. Under proper anesthesia with 2% to 3% isoflurane, a small incision was made in the atlanto-occipital membrane of the cisterna magna, and a PE-10 catheter (∼8.0 cm) was inserted to the lumbar spinal cord level.^13^ Animals presenting signs of neurological or motor dysfunction within 5 days after surgery were humanely destroyed. Rats with pain thresholds 20% below the average of all rats were excluded before the following experiments started.

### Establishment of PINP model

The PINP model was performed as previously described.^14^ Pharmaceutical-grade paclitaxel was diluted with sterile saline from the original stock concentration of 6 mg/mL (in 1:1 ethanol: Cremophor EL). After baseline measurement, rats were intraperitoneally injected with paclitaxel (8 mg/kg) for 3 days (D1, D4, and D7). The cumulative dose of paclitaxel was 24 mg/kg. Control rats were treated with the same contractions of ethanol and Cremophor EL but without paclitaxel. No spontaneous abnormal behaviors were observed during or after control or paclitaxel treatment.

### Assessment of mechanical allodynia

A double-blind match was performed before the tests. Mechanical allodynia was assessed as previously described.^15^ The 2390 Electronic von Frey Anesthesiometer (IITC Life Science, USA) with a cut off of 50 g was used for the measurements. After acclimation in behavioral chambers for 15 min, the hind plantars of both sides were measured. Mechanical allodynia was indicated by a significant decrease in the mean *paw withdrawal thresholds* (PWTs). The nociceptive PWT (g) was defined as the force (g) which caused the rat withdrew its paw. The PWT (g) of the rat was defined as the average PWT (g) of both sides. Each measurement was triplicated with 5 min interval.

### Enzyme-linked immunosorbent assay

Under deep anesthesia with sodium pentobarbital (100 mg/kg), L4-L6 spinal cords of each rat were rapidly isolated and separated into several parts on day 15. Parts of the spinal cords were homogenized in RIPA buffer with phenylmethylsulfonyl fluoride at 4°C. After 4000 r/min centrifugation for 15 min at 4°C, supernatant was determined through bicinchoninic acid method. After being equilibrated, the samples were used for ELISA. Proinflammatory factors (interleukin [IL-1β], interleukin [IL-6], and tumor necrosis factor [TNF-α]) were measured by rat-specific ELISA kits (Kangwei Bio-tech., China) according to the manufacturer’s instructions.

### Assessment of NAD content

The content of NAD was detected according to previous study.^12^ Same mass of the spinal cords among the groups lysed in 200 ml HClO_4_ (1.0 M) containing 3.2 nmol [^18^O] NAD. The supernatant was collected after centrifugation at 12,000 r/min for 10 min at room temperature, neutralized with NaOH, and injected on a C-18 semipreparative column. NAD fractions were collected according to the retention time of authentic standards, lyophilized, and redissolved in 50% acetonitrile. Matrix-assisted laser desorption ionization-mass spectroscopy (positive mode) was used to detect NAD signal. Appropriate blanks and controls were also analyzed together.

### Immunoblot

After being determined through bicinchoninic acid method, a portion of the samples was mixed with sodium dodecyl sulfate loading buffer and boiled at 99°C for 5 min. Then, the samples were separated by sodium dodecyl sulfate polyacrylamide gel electrophoresis, transferred to 0.45 nm polyvinylidene difluoride membranes (Millipore, USA), and incubated overnight with indicated antibodies. GAPDH (Proteintech, China, 1:5000), H4-k16Ac, H4, NF-κB(p65), p-p65 (Cell Signaling Technology, USA, 1:1000), glial fibrillary acidic protein (GFAP), and SIRT1 (Abcam, UK, 1:1000) were used in this study. After incubation with secondary antibodies, the proteins were detected by enhanced chemiluminescence.

### Immunofluorescence

Parts of spinal cords were immersed in 4% paraformaldehyde for 8 h and 30% sucrose overnight at 4°C. After embedded and sliced, the sections were incubated with 0.3% Triton X-100 for 20 min, 5% donkey serum for an hour, and primary antibody in 1% donkey serum overnight at 4°C. Rabbit anti-NF-κB(p65) antibody (Cell Signaling Technology, USA, 1:200) and goat anti-GFAP antibody(Abcam, UK, 1:200) were used. After being washed again, sections were respectively treated with donkey anti-rabbit IgG Dylight488 and goat IgG Dylight594 (1:200, Jackson ImmunoResearch Laboratories, USA). Then, the sections for NF-κB(p65) detection were treated with DAPI for 10 min. Then, the sections were washed with phosphate-buffered saline for three times. All sections were visualized with fluorescence microscope (Leica DM500B, Wetzlar, Germany).

## Statistical analysis

Data presented in this study were expressed as Mean ± *SEM*. The data were analyzed by SPSS (version 17.0) (SPSS Inc., Chicago, IL). MT baselines in part one and two were analyzed by one way-analysis of variance. Data of the behavior tests after paclitaxel treatment in part one and two were analyzed by one analysis of variance followed by least significant difference comparisons. Comparisons among multiple groups in western blot and ELISA results in part one and two were analyzed by with Student–Newman–Keuls tests post-hoc analysis. Significant difference was considered as *P* < 0.05.

## Results

### Effect of icariin on paclitaxel-induced mechanical allodynia in rats

**Figure 1.**
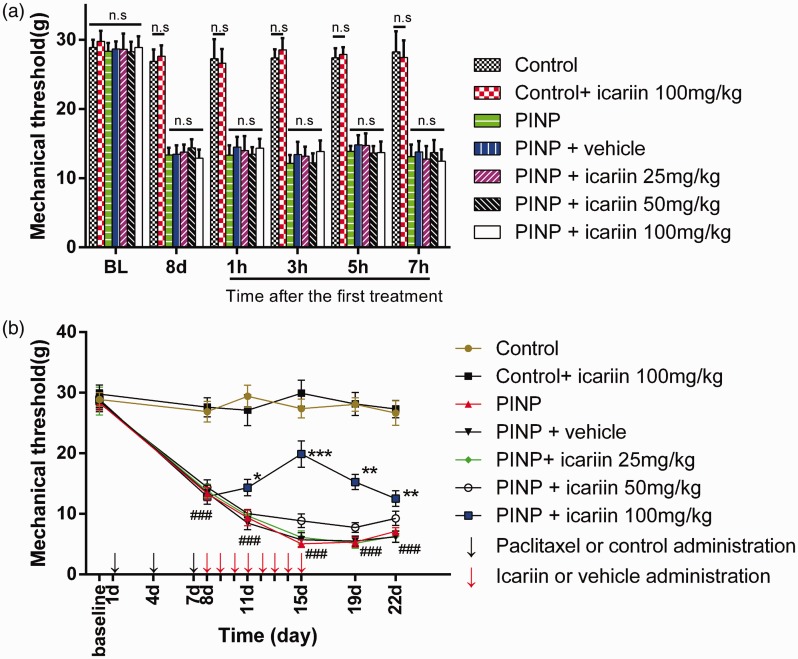
Effect of icariin on paclitaxel-induced mechanical allodynia on the short and long term. (a) A single administration of icariin at the dosage of 25 mg/kg, 50 mg/kg, and 100 mg/kg did not alter paclitaxel-induced mechanical allodynia in 7 h on day 7. (b) Daily treatment with icariin 100 mg/kg from day 7 to day 14 reversed paclitaxel-induced mechanical allodynia since day 10 (*P<0.05, ^**^P<0.01, ^***^P<0.001, vs. PINP group). Icariin 25 mg/kg or 50 mg/kg treatment did not significantly alleviate paclitaxel-induced mechanical allodynia (*P*>0.05). Data were expressed as mean ± *SEM* (*n*=6). PINP: paclitaxel-induced neuropathic pain.

Results showed no significant difference on MTs baseline among the groups (*P*≥0.05). Control treatment did not affect the MTs in the short (Figure 1(a), *P*≥0.05) and long terms (Figure 1(b), *P*≥0.05). In addition, 100 mg/kg icariin treatment did not influence the MTs in control rats (*P*≥0.05). Paclitaxel treatment induced a decrease of MTs by about 52.9% in day 8. The MTs reached a lowest peak on day 15, which decreased by about 82.1%. Vehicle treatment did not significantly relieve paclitaxel-induced mechanical allodynia in short term (Figure 1(a), *P*≥0.05) or long terms (Figure 1(a), *P*≥0.05), compared to PINP group. Daily oral 25 mg/kg or 50 mg/kg icariin did not significantly relieve the paclitaxel-induced mechanical allodynia in short (Figure 1(a), *P*_25 mg/kg_≥0.05; *P*_50 mg/kg_≥0.05) or long terms (Figure 1(b), *P*_25 mg/kg_≥0.05; *P*_50 mg/kg_≥0.05), compared to PINP group; 100 mg/kg icariin treatment did not relieve paclitaxel-induced mechanical allodynia in the short term (Figure 1(a)*, P*≥0.05). However, icariin 100 mg/kg treatment significantly alleviated paclitaxel-induced mechanical allodynia in the long term (Figure 1(b), *F*(1, 60)=43.477, *P*<0.001).

### Effect of icariin on expressions of TNF-α, IL-1β, and IL-6 in the spinal cords

**Figure 2.**
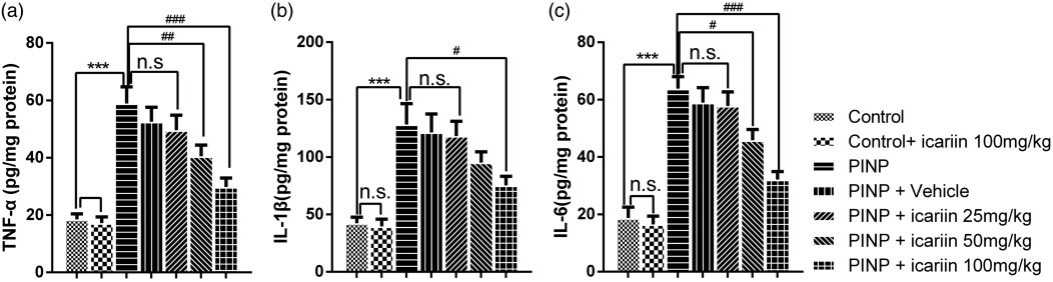
Effect of icariin on paclitaxel-induced production of TNF-α (a), IL-1β (b), and IL-6 (c) in the spinal cords by ELISA. Paclitaxel treatment significantly induced the production of TNF-α, IL-1β, and IL-6 in the spinal cord (^***^P<0.001 vs. control group). Icariin 100 mg/kg treatment significantly suppressed paclitaxel-induced upregulation of TNF-α, IL-1β, and IL-6 (^#^*P*<0.05, ^###^*P*<0.001, vs. PINP group). Icariin 50 mg/kg treatment significantly inhibited paclitaxel-induced upregulation of TNF-α and IL-6 but not IL-1β (^#^*P*<0.05, ^##^*P*<0.01, vs. PINP group). Data were expressed as mean ± *SEM* (*n*=6). PINP: paclitaxel-induced neuropathic pain.

Expressions of TNF-α, IL-1β, and IL-6 in the spinal cords were measured by ELISA (Figure 2). Results showed that paclitaxel administration significantly upregulated the expressions of TNF-α, IL-1β, and IL-6 compared to control group (*P*_TNF-α_<0.001, *P*_IL-1β_<0.001, *P*_IL-6_ <0.001). Icariin 100 mg/kg treatment did not influence the contents of IL-1β, IL-6, and TNF-α in control rats (*P*≥0.05). Vehicle treatment did not influence the contents of IL-1β, IL-6, and TNF-α in PINP rats (*P*≥0.05). Icariin 25 mg/kg treatment did not affect the expressions of IL-1β, IL-6, and TNF-α in PINP rats, neither (*P*≥0.05). Icariin 50 mg/kg administration alleviated paclitaxel-induced production of TNF-α and IL-6 but not IL-1β (*P*≥0.05). However, Icariin 100 mg/kg administration completely suppressed the expressions of TNF-α, IL-1β, and IL-6 in PINP rats (*P*_TNF-α_<0.001, *P*_IL-1β_=0.024, *P*_IL-6_ <0.001).

### Effect of icariin on astrocyte activation in the spinal cords

**Figure 3.**
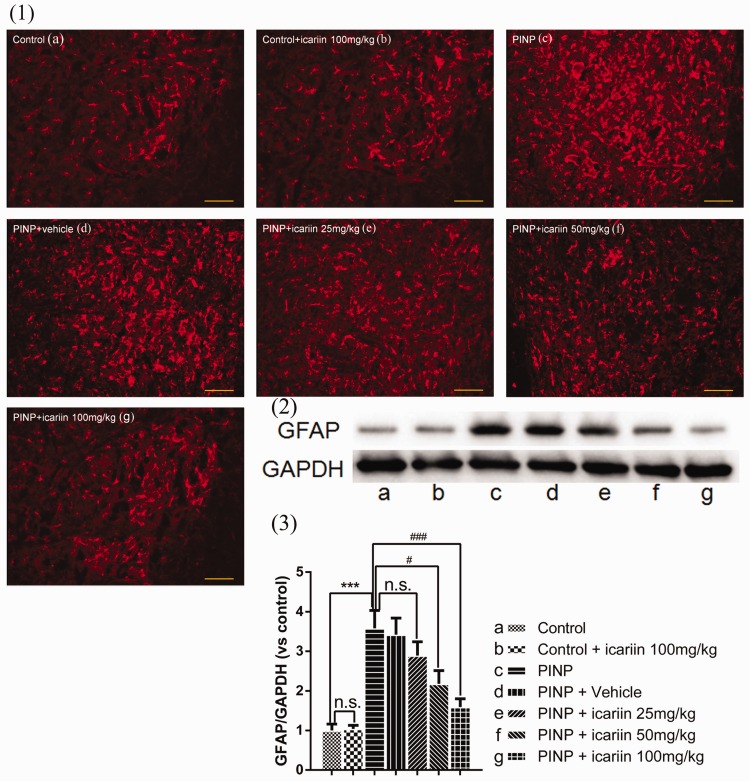
Effect of icariin on paclitaxel-induced astrocyte activation in the spinal cords. (1) Immunofluorescence results indicating that paclitaxel treatment significantly activated astrocytes in the spinal cord. Icariin 100 mg/kg treatment significantly reversed paclitaxel-induced astrocyte activation. For better qualification, we examined the expression of GFAP in the spinal cord (2). Results showing the same trend (3). ^***^P<0.001 vs. control group, ^#^*P*<0.05, ^###^*P*<0.001, vs. PINP group. Data were expressed as mean ± *SEM* (*n*=6). PINP: paclitaxel-induced neuropathic pain.

Activated astrocytes in the spinal cords were measured by immunofluorescence (Figure 3(1)) and immunoblot of GFAP (Figure 3(2) and (3)). Immunofluorescence results showed that icariin 100 mg/kg treatment did not affect the number of activated astrocytes in control rats. Paclitaxel treatment significantly induced astrocytes activation in the spinal cords. This effect was suppressed by icariin 100 mg/kg treatment. To better qualify the results, expression of GFAP in L4-L6 spinal cords was examined by immunoblot. Results showed that no significant difference was found between control and control+icariin 100 mg/kg group (*P*≥0.05). Vehicle treatment also did not significantly influence GFAP expression in PINP rats (*P*≥0.05). Icariin 25 mg/kg treatment did not significantly decrease GFAP expression, neither (*P*≥0.05). However, icariin 50 mg/kg administration significantly inhibited paclitaxel-induced upregulation of GFAP (*P*=0.017). This effect was more evident in PINP+icariin 100 mg/kg group.

### Effect of icariin on spinal NF-κB(p65) activation in rats

**Figure 4.**
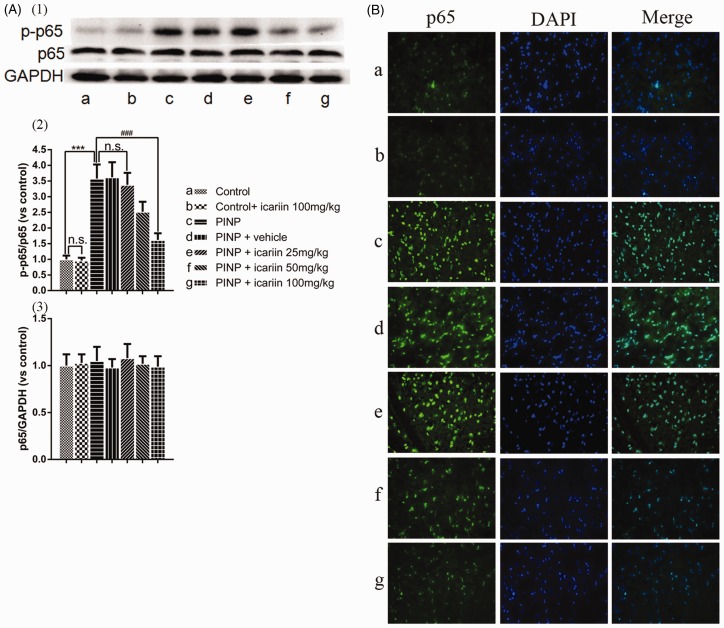
Effect of icariin on paclitaxel-induced NF-κB(p65) activation in the spinal cords. (A) Western blot results showing that paclitaxel treatment induced NF-κB(p65) phosphorylation without total NF-κB(p65) upregulation in the spinal cords ((2) and (3)). Icariin 100 mg/kg treatment inhibited paclitaxel-induced NF-κB(p65) phosphorylation. ^***^P<0.001 vs. control group, ^###^*P*<0.001, vs. PINP group. (B) Immunohistochemistry results indicating that paclitaxel treatment induced p65 nuclear translocation in the spinal dorsal horn. However, this effect was significantly suppressed by icariin 100 mg/kg treatment. Data were expressed as mean ± *SEM* (*n*=6). PINP: paclitaxel-induced neuropathic pain.

Spinal NF-κB(p65) activation was measured though detection of NF-κB(p65) phosphorylation (Figure 4(a)) and intracellular distribution of NF-κB(p65) in the spinal dorsal horn (Figure 4(b)). Immunoblot results showed that paclitaxel significantly induced NF-κB(p65) phosphorylation, compared to control rats (*P*<0.001). However, icariin 100 mg/kg treatment showed no influence on NF-κB(p65) phosphorylation in control rats. Vehicle treatment did not alter paclitaxel-induced NF-κB(p65) phosphorylation, neither. Icariin 100 mg/kg treatment significantly inhibited paclitaxel-induced NF-κB(p65) phosphorylation but not icariin 25 mg/kg or 50 mg/kg treatment (*P*_25 mg/kg_≥0.05, *P*_50 mg/kg_≥0.05, *P*_100 mg/kg_ <0.001). No significant difference was found among the groups on total NF-κB(p65) expression (*P*≥0.05). Further, results of intracellular NF-κB(p65) distribution indicated that paclitaxel induced NF-κB(p65) nuclear translocation in the spinal dorsal horn. However, this effect was significantly suppressed by icariin 100 mg/kg treatment.

### Effect of icariin on spinal SIRT1expression, its target H4-K16 acetylation (H4-K16Ac) and NAD content in the spinal cord

**Figure 5.**
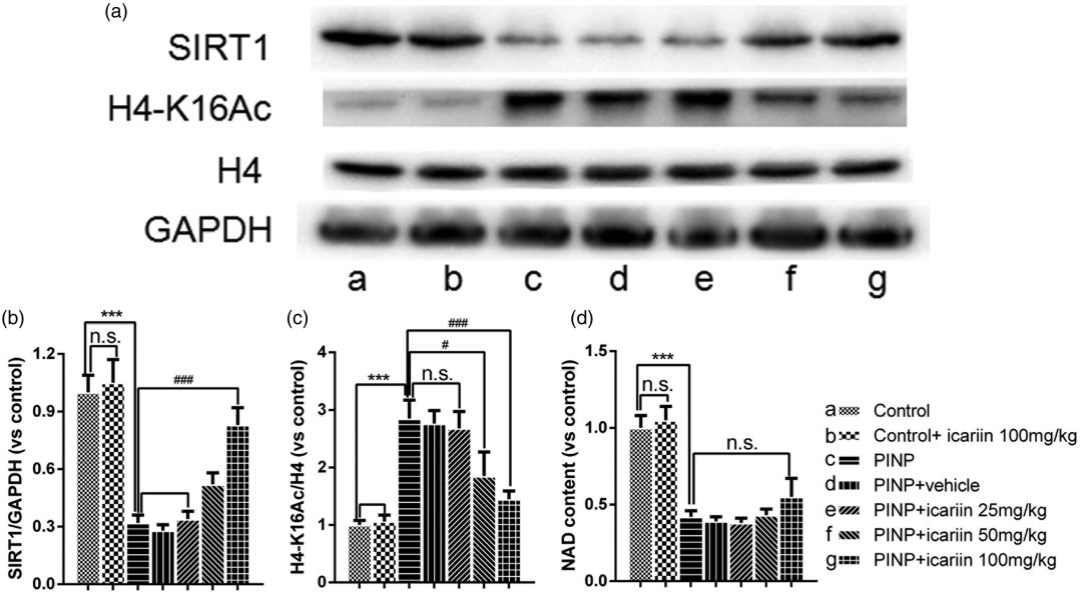
Effect of icariin on spinal SIRT1 expression, H4-K16 acetylation (H4-K16Ac), and NAD content in the spinal cord. Paclitaxel treatment significantly induced SIRT1 downregulation, H4-K16 acetylation, and the decrease of NAD content in the spinal cord. Icariin 100 mg/kg treatment significantly reversed paclitaxel-induced SIRT1 downregulation, H4-K16 acetylation but not the decrease of NAD content. Icariin 50 mg/kg treatment suppressed paclitaxel-induced H4-K16 acetylation but not SIRT1 downregulation or the decrease of NAD content. ^***^P<0.001 vs. control group, ^#^*P*<0.05, ^###^*P*<0.001, vs. PINP group. Data were expressed as mean ± *SEM* (*n*=6). PINP: paclitaxel-induced neuropathic pain.

Results showed that paclitaxel treatment significantly decreased expression of SIRT1 in the spinal cord (Figure 5, *P*<0.001). Icariin 100 mg/kg treatment blocked the effect of paclitaxel on spinal SIRT1 expression (*P*<0.001). Vehicle, icariin 25 mg/kg, or icariin 50 mg/kg did not significantly affect paclitaxel-induced decrease of SIRT1 expression (*P*≥0.05). Neither significant difference on SIRT1 expression was found between control and control+icariin 100 mg/kg group (*P*≥0.05). To investigate the SIRT1 deacetylase activity among the groups, immunoreactive H4-K16Ac expression was detected. Results indicated that paclitaxel treatment significantly upregulated H4-k16Ac content (*P*<0.001). This meant that paclitaxel treatment inhibited SIRT1 deacetylase activity in the spinal cord. Icariin 50 mg/kg or 100 mg/kg significantly reversed paclitaxel-induced upregulation of H4-k16Ac content (*P*_50 mg/kg_=0.042, *P*_50 mg/kg_=0.004). Vehicle or icariin 25 mg/kg treatment did not significantly influence expression of H4-k16Ac content in PINP rats (*P*≥0.05). Neither significant difference on H4-k16Ac content was found between control and control+icariin 100 mg/kg group (*P*≥0.05). Further, we detected the NAD content in the spinal cords. Results showed that paclitaxel treatment significantly decreased NAD content in the spinal cord (*P*<0.001). However, icariin 100 mg/kg treatment did not influence the NAD content in control (*P*≥0.05) or PINP rats (*P*≥0.05). These meant that icariin treatment increased SIRT1 expression and activity in an NAD-independent manner.

### Reversal of icariin-induced effects by EX527, a SIRT1 inhibitor

**Figure 6.**
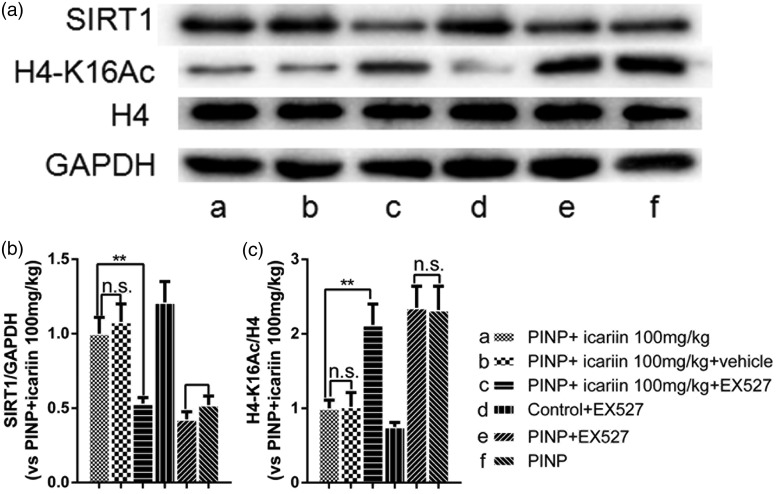
Blockage of icariin-induced SIRT1 activation by EX527, a SIRT1 inhibitor. Intrathecal EX527 treatment completely inhibited icariin-induced SIRT1 upregulation and H4-K16 acetylation in PINP+icariin rats. However, intrathecal EX527 treatment did not alter SIRT1 expression and H4 acetylation in control rats (data not shown). Nor did EX527 treatment alter SIRT1 expression and H4 acetylation in PINP rats. ^**^P<0.01 vs. PINP+icariin group. Data were expressed as mean ± *SEM* (*n*=6). PINP: paclitaxel-induced neuropathic pain.

**Figure 7.**
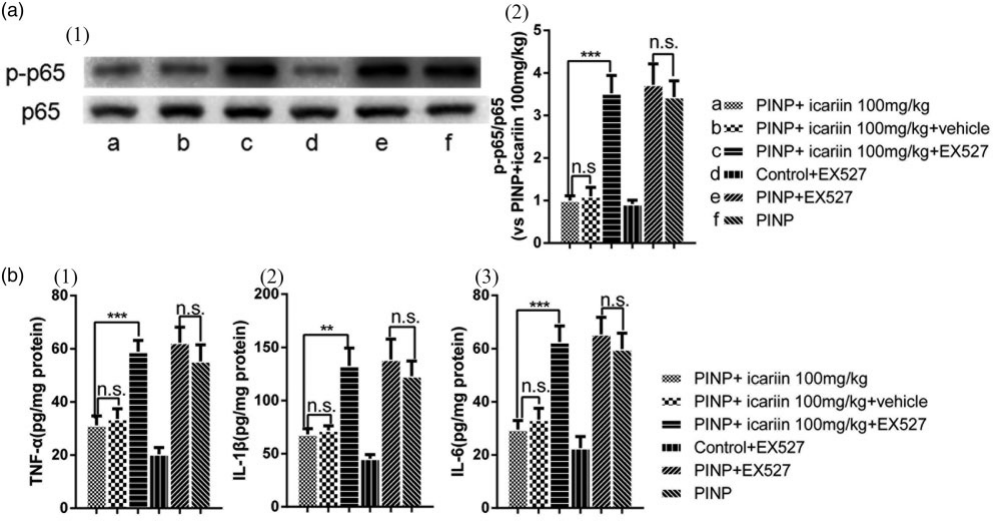
Inhibition of icariin-induced anti-neuroinflammation effects by EX527. (a) Icariin-induced suppression of NF-κB(p65) phosphorylation was completely reversed by EX527 treatment. (b) Icariin-induced downregulation of TNF-α, IL-1β, and IL-6 in the spinal cords was completely inhibited by EX527 treatment. However, intrathecal EX527 treatment did not alter NF-κB(p65) phosphorylation and production of TNF-α, IL-1β, and IL-6 in control rats (data not shown). Nor did EX527 treatment alter NF-κB(p65) phosphorylation and production of TNF-α, IL-1β, and IL-6 in PINP rats. ^**^P＜0.01, ^***^P＜0.001, vs. PINP+icariin group. Data were expressed as mean ± *SEM* (*n*=6). PINP: paclitaxel-induced neuropathic pain.

To investigate the role of SIRT1 on icariin-induced effects, EX527, a selective SIRT1 inhibitor, was used. First, we detected EX527 on expression of SIRT1 and H4-k16Ac among the groups (Figure 6). Results in part two showed that EX527 significantly inhibited icariin-induced SIRT1 upregulation and H4-k16Ac downregulation in PINP rats (Figure 7, *P*_SIRT1_=0.002, *P*_H4-k16Ac_ =0.005, vs. PINP+ icariin 100 mg/kg group). While vehicle treatment did not affect the expression of SIRT1 and H4-k16Ac in the spinal cord (*P*_SIRT1_=0.555, *P*_H4-k16Ac_≥0.05). EX527 treatment in control rats also showed no significant difference on the expression of SIRT1 and H4-k16Ac, compared to control group in part one (data not shown). EX527 treatment did not significantly influence SIRT1 and H4-k16Ac expression in PINP rats, neither (*P*_SIRT1_≥0.05, *P*_H4-k16Ac_ ≥0.05).

Second, we investigated the effect of EX527 on expression of pro-inflammatory factors (IL-1β, IL-6, and TNF) and NF-κB(p65) activation in icariin-treated PINP rats (Figure 7). Results showed that EX527 abolished icariin-induced inhibition of p65 phosphorylation in PINP rats (*P*<0.001). EX527 also reversed icariin-induced inhibition on the production of TNF-α, IL-1β, and IL-6 in PINP rats (*P*_TNF-α_<0.001, *P*_IL-1β_=0.005, *P*_IL-6_ <0.001). However, no difference was found compared PINP +icariin 100 mg/kg with vehicle group (*P*≥0.05). No significant difference was found between PINP+EX527 and PINP group (*P*≥0.05). Neither significant difference was found between control+EX527 group and control group in part one (data not shown).

**Figure 8.**
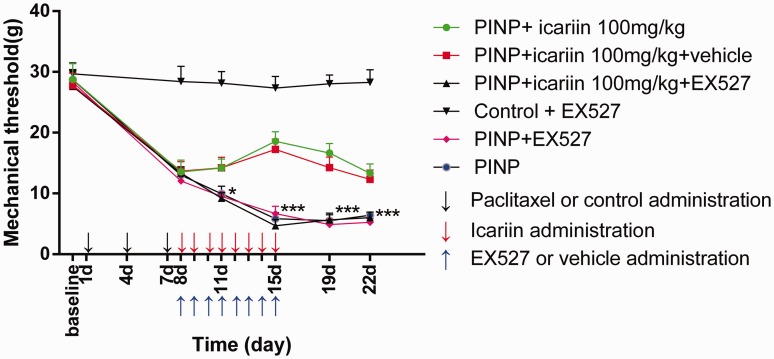
Reversal of icariin-induced anti-allodynia effect by EX527. EX527 treatment completely abrogated icariin-induced anti-allodynia effects in PINP rats. However, EX527 treatment in control rats did not significantly influence the mechanical thresholds. EX527 treatment alone did not influence the mechanical thresholds in PINP rats. ^###^*P*＜0.001 vs. PINP+icariin group. Data were expressed as mean ± *SEM* (*n*=6). PINP: paclitaxel-induced neuropathic pain.

Third, we investigated the impact of EX527 on icariin-induced anti-allodynia effect in PINP rats (Figure 8). No significant difference was found before or after EX527 treatment in control rats (*P*≥0.05). No significant difference was found comparing PINP with PINP+EX527 group (*P*≥0.05). Neither significant difference was found between PINP+ icariin group and PINP + icariin+ vehicle group (*P*≥0.05). However, EX527 completely abrogated icariin-induced anti-allodynia effect in PINP rats (*F*(1, 60)=22.506, *P*<0.001). This meant that SIRT1 was involved in the effects of icariin in PINP rats.

## Discussion

It was widely accepted that neuroinflammation played a role in central sensitization of the chemotherapeutic drug-induced painful neuropathy. In this study, we demonstrated that icariin, a compound from Epimedium that possessed anti-neuroinflammation, anti-cancer, and male enhancement effect, suppressed paclitaxel-induced neuroinflammation and mechanical allodynia in rats. Icariin alleviated paclitaxel-induced SIRT1 downregulation and H4-16K acetylation. However, blocking SIRT1 by EX527 completely reversed icariin-induced H4 deacetylation, anti-neuroinflammation, and anti-allodynia effects. These results suggested icariin-induced anti-allodynia effect in PINP rats in a SIRT1-dependent manner.

SIRT1 is a histone deacetylase that regulates oxidative respiration, inflammatory responses, and various physical conditions. SIRT1 activation was reported to alleviate pain behaviors in neuropathic pain. Yin et al.^16^ first reported that resveratrol, a SIRT1 agonist, attenuated neuropathic pain-like behaviors. Spinal SIRT1 activation attenuated spinal H4-K16 acetylation and showed anti-nociceptive effect in neuropathic pain.^12^ Zhou et al.^17^ reported that SIRT1 activation induced epigenetic regulation of mGluR1/5 expressions in diabetic neuropathic pain. In our results, icariin treatment significantly reversed paclitaxel-induced SIRT1 downregulation and H4-K16 acetylation. However, icariin treatment did not influence the NAD^+^ content in our study. Furthermore, EX527 blocked icariin-induced SIRT1 upregulation and H4-K16 deacetylation. This meant that SIRT1 played an important role in icariin-induced effects.

Various studies reported a close relationship between NAD^+^ content and SIRT1.^18,19^ In normal conditions, SIRT1 induced AMPK (adenosine 5′-monophosphate (AMP)-activated protein kinase) activation via LKB1 (Liver kinase B1) deacetylation, which subsequently activated AMPK.^20^ AMPK promoted cellular NAD^+^ synthesis by targeting intra-cellular nicotinamide phosphoribosyltransferase, which was required for SIRT1 activity.^21^ In our study, paclitaxel significantly decreased NAD^+^ content and SIRT1 expression in the spinal cord. However, daily 100 mg/kg icariin treatment induced SIRT1 upregulation and the deacetylase activity without affecting the NAD^+^ content in PINP rats. This was unconventional to previous studies. However, studies reported that PARP1/2 (Poly(ADP-ribose) polymerase 1/2), an enzyme that promoted NAD^+^ degeneration, promoted the development of chemotherapy-induced peripheral neuropathy.^22,23^ Whether PARP1/2 induced inhibition effect on NAD^+^ content in icariin-treated PINP rats was unknown. Besides, Gerhart-Hines et al.^24^ uncover a rapidly acting mechanism of SIRT1 regulation through β-adrenergic activation of the cAMP/PKA pathway rapidly increases SIRT1 activity in a NAD+-independent fashion.^19^ Whether icariin induced SIRT1 activation in a NAD+ dependent manner still needed more researches.

NF-κB(p65), a widely expressed transcriptional factor, controlled the expression of many pro-inflammatory factors. Under normal condition, p65 was retained in the cytoplasm by inhibitor of NF-κB (IκB). Once activated, p65 was translocated to the nucleus and recruited to the targeted genes. p65 phosphorylation significantly upregulated the transcriptional activity. Li et al.^3^ reported that activation of NF-κB(p65) also increased H4 acetylation and epigenetic regulation of the production of pro-inflammatory factors. Our previous study reported that activation of SIRT1 induced downregulation of NF-κB(p65) signaling.^25^ In this study, activation of SIRT1 by icariin reversed paclitaxel-induced NF-κB(p65) phosphorylation and nucleus translocation. Icariin treatment also blocked the production of TNF-α, IL-1β, and IL-6. This was also consistent with the previous studies.

Studies were reported that spinal astrocytes but not microglia contributed to the formation of central sensitization and maintenance of paclitaxel-induced mechanical allodynia.^26^ Spinal-activated astrocytes often produced a variety of pro-inflammatory factors including TNF-α, IL-1β, and IL-6. These factors promoted neuron activity and suppressed synaptic transmission in the spinal cord.^27,28^ However, Zhang et al.^26^ reported that detection of TNF-α, IL-1β, and IL-6 in the spinal dorsal horn by reverse transcription polymerase chain reaction showed no significant increase in paclitaxel-treated rats. In our results, paclitaxel treatment significantly induced astrocytes activation as well as production of TNF-α, IL-1β, and IL-6 in the spinal cord. We proposed that this might be the results of different methods for the targets. Icariin treatment suppressed astrocytes activation as well as expression of production of TNF-α, IL-1β, and IL-6. However, this was completely blocked by EX527. This was consist with the results of previous studies.^9^

There were some limitations or difference in this study. First, most studies used the cumulative dose of paclitaxel at 4 or 8 mg/kg for the establishment of PINP pain model. However, the cumulative dose of paclitaxel at 24 mg/kg was used in this study. Since the recommended dosage of paclitaxel for cancer was 135 to 175 mg/m^2^ in clinical treatment. According to Meeh-Rubner formula,^29^ the dosage of paclitaxel for rats was 22.5 to 29.5 mg/kg. To reduce variation between the experiments and the clinic, we used the cumulative dose of paclitaxel at 24 mg/kg for the model. By the way, Li et al.^3^ also used the cumulative dose of 24 mg/kg for PINP model as we did. During the study, no significant difference was found among the groups on some basic data such as bodyweight and locomotor activity (data not shown). Second, icariin at 50 mg/kg was effective for inhibition of paclitaxel-induced upregulation of inflammatory cytokines, activation of astrocytes, activation of NF-κB, and the increase in H4 acetylation but cannot affect mechanical allodynia induced by paclitaxel. This might be a limitation in the study, due to the small sample size, the results and conclusions would have thus been sensitive to even a single value changing.

In summary, icariin suppressed paclitaxel-induced neuroinflammation and mechanical allodynia in a SIRT1-dependent manner. Icariin could be a potential agent for the treatment of PINP.

## Highlights

Icariin dosage-dependently alleviated paclitaxel-induced mechanical allodynia in rats.

Icariin dosage-dependently suppressed paclitaxel-induced neuroinflammation in L4-L6 spinal cords.

Icariin dosage-dependently reversed paclitaxel-induced SIRT1 downregulation and H4 acetylation in the spinal cord but not the decrease of NAD^+^ content.

EX527 inhibited icariin-induced effects in PINP rats.

Icariin could be a potential therapeutic agent for paclitaxel-induced neuropathic pain.
